# Dynamic causal models of steady-state responses

**DOI:** 10.1016/j.neuroimage.2008.09.048

**Published:** 2009-02-01

**Authors:** R.J. Moran, K.E. Stephan, T. Seidenbecher, H.-C. Pape, R.J. Dolan, K.J. Friston

**Affiliations:** a Wellcome Trust Centre for Neuroimaging, Institute of Ne.urology, University College London, 12 Queen Square, London WC1N 3BG, UK; b Laboratory for Social and Neural Systems Research, Institute for Empirical Research in Economics, University of Zurich, Switzerland; c Institute of Physiology, University of Münster, Germany

**Keywords:** Frequency domain electrophysiology, Bayesian inversion, Cross-spectral densities, DCM, Fear conditioning, Hippocampus, Amygdala

## Abstract

In this paper, we describe a dynamic causal model (DCM) of steady-state responses in electrophysiological data that are summarised in terms of their cross-spectral density. These spectral data-features are generated by a biologically plausible, neural-mass model of coupled electromagnetic sources; where each source comprises three sub-populations. Under linearity and stationarity assumptions, the model's biophysical parameters (e.g., post-synaptic receptor density and time constants) prescribe the cross-spectral density of responses measured directly (e.g., local field potentials) or indirectly through some lead-field (e.g., electroencephalographic and magnetoencephalographic data). Inversion of the ensuing DCM provides conditional probabilities on the synaptic parameters of intrinsic and extrinsic connections in the underlying neuronal network. This means we can make inferences about synaptic physiology, as well as changes induced by pharmacological or behavioural manipulations, using the cross-spectral density of invasive or non-invasive electrophysiological recordings. In this paper, we focus on the form of the model, its inversion and validation using synthetic and real data. We conclude with an illustrative application to multi-channel local field potential data acquired during a learning experiment in mice.

## Introduction

This paper is concerned with modelling steady-state or (quasi) stationary responses recorded electrophysiologically using invasive or non-invasive techniques. Critically, the models are parameterised in terms of neurophysiologically meaningful parameters, describing the physiology and connectivity of coupled neuronal populations subtending observed responses. The model generates or predicts the cross-spectral density of observed responses, which are a simple but comprehensive summary of steady-state dynamics under linearity and stationarity assumptions. Furthermore, these cross-spectral features can be extracted quickly and simply from empirical data. In this paper, we describe the model and its inversion, with a focus on system identifiability and the validity of the proposed approach. This method is demonstrated using local field potentials (LFP) recorded from Pavlovian fear conditioned mice. In subsequent papers, we will apply the model to LFP data recorded during pharmacological experiments.

The approach described below represents the denouement of previous work on dynamic causal modelling of spectral responses. In Moran et al., (2007), we described how neural-mass models, used originally to model evoked responses in the electroencephalogram (EEG) and magnetoencephalogram (MEG) (David et al., 2003, 2005; Kiebel et al., 2007), could also model spectral responses as recorded by LFPs. This work focussed on linear systems analysis and structural stability, in relation to model parameters. We then provided a face validation of the basic idea, using single-channel local field potentials recorded from two groups of rats. These groups expressed different glutamatergic neurotransmitter function, as verified with microdialysis (Moran et al., 2008). Using the model, we were able to recover the anticipated changes in synaptic function.

Here, we generalise this approach to provide a full dynamic causal model (DCM) of coupled neuronal sources, where the ensuing network generates electrophysiological responses that are observed directly or indirectly. This generalisation rests on two key advances. First, we model not just the spectral responses from each electromagnetic source but the cross-spectral density among sources. This enables us to predict the cross-spectral density in multi-channel data, even if it has been recorded non-invasively through, for example, scalp electrodes. Second, in our previous work we made the simplifying assumption that the neuronal innovations (i.e. the baseline cortical activity) driving spectral responses were white (i.e., had uniform spectral power). In this work, we relax this assumption and estimate, from the data, the spectral form of these innovations, using a more plausible mixture of white and pink (1/*f*) components.

This paper comprises three sections. In the first, we describe the DCM, the cross-spectral data-features generated by the model and model inversion given these features. In the second section, we address the face validity of the model, using synthetic data to establish that both the form of the model and its key parameters can be recovered in terms of conditional probability densities. The parameters we look at are those that determine post-synaptic sensitivity to glutamate from extrinsic and intrinsic afferents. In the final section, we repeat the analysis of synthetic data using multi-channel LFP data from mice, acquired during cued recall of a conditioned fear memory. This section tries to establish the construct validity of DCM in relation to the previous analyses of functional connectivity using cross-correlogram analysis. These show an increase in the coupling between the hippocampus and amygdala using responses induced by conditioned fear-stimuli. We try to replicate this finding and, critically, extend it to establish the changes in directed connections that mediate this increased coupling.

## The dynamic causal model

In this section, we describe the model of cross-spectral density responses. Much of this material is based on linear systems theory and the differential equations that constitute our neural-mass model of underlying dynamics. We will use a tutorial style and refer interested readers to appendices and previous descriptions of the neural-mass model for details. We first consider the generative model for cross-spectral density and then describe how these cross-spectral features are evaluated. Finally, we review model inversion and inference.

### A generative model for cross-spectral density

Under stationarity assumptions, one can summarize arbitrarily long electrophysiological recordings from multi-channel data in terms of cross-spectral density matrices, *g*(ω)_*c*_ at frequency ω (radians per second). Heuristically, these can be considered as a covariance matrix at each frequency of interest. As such, these second-order data-features specify, completely, the second-order moments of the data under Gaussian assumptions. Cross-spectral density is useful because it represents the important information, in long time-series, compactly. Furthermore, it brings our data modelling into the domain of conventional spectral analysis and linear systems theory. The use of linear systems theory to derive the predicted spectral response from a non-linear dynamical system assumes that changes in the (neuronal) states of the system can be approximated with small perturbations around some fixed-point. This assumption depends on the experimental design and is more easily motivated when data are harvested during periods of limited perturbations to the subject's neuronal state. In short, we discount the possibility of phase-transitions and bifurcations (e.g., oscillatory dynamics) due to the non-linear properties of cortical macrocolumns (e.g. Breakspear et al., 2006).

#### The neural mass model

**Fig. 1:**
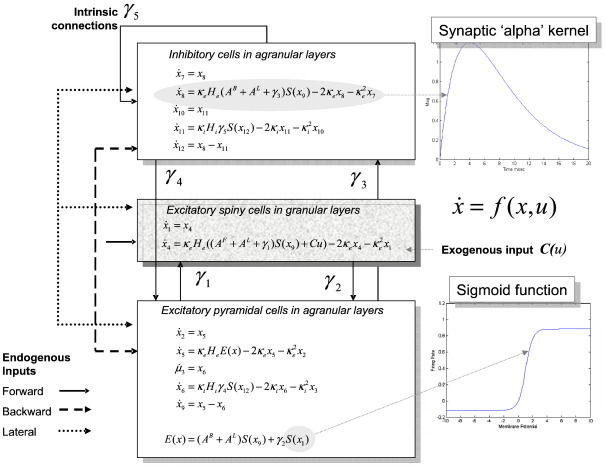
Schematic of the source model with intrinsic connections. This schematic includes the differential equations describing the motion of hidden electrophysiological states. Each source is modelled with three subpopulations (pyramidal, spiny-stellate and inhibitory interneurons) as described in (Jansen and Rit, 1995). In this figure these subpopulations have been assigned to granular and agranular cortical layers, which receive forward, backward and lateral connections from extrinsic sources in the network.

The underlying dynamic causal model is defined by the equations of motion $\dot{x}(t)=f\left(x\text{,}u\right)$ at the neuronal level. In this context, they correspond to a neural-mass model that has been used extensively in the causal modelling of EEG and MEG data and has been described previously for modelling spectral responses (Moran et al., 2007, 2008). This model ascribes three sub-populations to each neuronal source, corresponding roughly to spiny stellate input cells, deep pyramidal output cells and inhibitory interneurons. Following standard neuroanatomic rules (Felleman and Van Essen 1991), we distinguish between forward connections (targeting spiny stellate cells), backward connections (targeting pyramidal cells and inhibitory interneurons with slower kinetics) and lateral connections (targeting all subpopulations); see Fig. 1 and Moran et al., (2007). Each neuronal source could be regarded as a three-layer structure, in which spiny stellate cells occupy the granular layer, while infragranular and supragranular layers contain both pyramidal cells and inhibitory interneurons.

Each subpopulation is modelled with pairs of first-order differential equations of the following form:

(1) $$\begin{array}{l} {\dot{x}}_{v}=x_{1}\\ {\dot{x}}_{I}=\kappa H\left(E(x)+C(u)\right)-2\kappa x_{1}-\kappa^{2}x_{v} \end{array}$$

The column vectors *x*_*v*_ and *x*_*I*_, correspond to the mean voltages and currents, where each element corresponds to the hidden state of the subpopulation at each source. These differential equations implement a convolution of a subpopulation's presynaptic input to produce a postsynaptic response. The output of each source is modelled as a mixture of the depolarisation of each subpopulation. Due to the orientation of deep pyramidal cell dendrites, tangential to the cortical surface, this population tends to dominate LFP recordings. We accommodate this by making the output of each source, *g*(*x*) a weighted mixture of *x*_*v*_ with weights of 60% for the pyramidal subpopulation and 20% for the others. The presynaptic input to each subpopulation comprises endogenous, *E*(*x*), and exogenous, *C*(*u*), components

#### Endogenous inputs

In a DCM comprising *s* sources, endogenous input *E*(*x*) is a weighted mixture of the mean firing rates in other subpopulations (see Fig. 1). These firing rates are a sigmoid activation function of depolarisation, which we approximate with a linear gain function; *S*(*x*_*i*_) = *Sx*_*i*_ ∈ ℜ^*s*^^x1^. Firing rates provide endogenous inputs from subpopulations that are intrinsic or extrinsic to the source. Subpopulations within each source are coupled by intrinsic connections, whose strengths are parameterised by *γ* = {*γ*_1_,…,*γ*_5_}. These endogenous intrinsic connections can arise from any subpopulation and present with small delays. Conversely, endogenous extrinsic connections arise only from the excitatory pyramidal cells of other sources and effect a longer delay than intrinsic connections. The strengths of these connections are parameterised by the forward, backward and lateral extrinsic connection matrices *A*^*F*^ ∈ ℜ^*s*^^xs^, *A*^*B*^ ∈ ℜ^*s*^^xs^ and *A*^*L*^ ∈ ℜ^*s*^^xs^ respectively. The postsynaptic efficacy of connections is encoded by the maximum amplitude of postsynaptic potentials *H*_*e,i*_ = *diag*(*H*_1_,…,*H*_s_) (note the subscripts in Fig. 1) and by the rate-constants of postsynaptic potentials, *κ* = *diag*(*κ*_1_,…,*κ*_s_) for each source. The rate-constants are lumped representations of passive membrane properties and other spatially distributed dynamics in the dendritic tree.

#### Exogenous inputs

Exogenous inputs *C*(*u*) = *Cu* are scaled by the exogenous input matrix *C* ∈ ℜ^*s*^^xs^ so that each source-specific innovation *u*(*t*) ∈ ℜ^*s*^^x1^ excites the spiny stellate subpopulation. We parameterise the spectral density of this exogenous input, *g*(ω)_*u*_, in terms of white (*α*) and pink (*β*) spectral components:

(2) $$g_{k}{\left(\omega \right)}_{u}=\alpha_{u}+\beta_{u}/\omega$$

#### Neuronal responses

The cross-spectral density is a description of the dependencies among the observed outputs of these neuronal sources. We will consider a linear mapping from *s* sources to *c* channels. In EEG and MEG this mapping is a lead-field or gain-matrix function, *L*(*θ*) ∈ ℜ^*c*^^x*s*^, of unknown spatial parameters, *θ*, such as source location and orientation. Generally, this function rests upon the solution of a well-posed electromagnetic forward model. For invasive LFP recordings that are obtained directly from the neuronal sources, this mapping is a leading diagonal gain-matrix, *L* = *diag*(*θ*_1_,...*θ*_*s*_) where the parameters model electrode-specific gains. The observed output at channel *i* is thus *S*_*i*_(*t*) = *L_i_g*(*x*), where *g*(*x*) is the source output (a mixture of depolarisations) and *L*_*i*_ represents the *i*-th lead-field or row of the gain-matrix. In other words, *L* = ℜ^1x*s*^ is the change in observed potential caused by changes in source activity. These observed outputs can now be used in a generative model of source cross-spectral measures.

#### Cross-spectral density

The neuronal model comprises a network of neuronal sources, each of which generates stationary time-series in a set of recording channels. These steady-state dynamics are expressed, in the frequency domain, as cross-spectral densities, g_*ij*_(*ω*), at radial frequencies ω, between channels *i* and *j*. Under linear systems theory, the cross-spectral density induced by the *k*-th input or innovation *u*_*k*_(*t*), is simply the cross-transfer function Γ*_ij_^k^*(*ω*) times the spectral density of that innovation, *g*_*k*_(*ω*)_*u*_. This transfer function is the cross-product of the Fourier transforms of the corresponding first-order kernels, *κ_i_^k^*(*t*) and *κ_i_^k^*(*t*) and in the case of *i* = *j* may be regarded as the modulation or self-transfer function).

(3) $$\begin{array}{l} \Gamma_{ij}^{k}(\omega )=\left|{\int \kappa_{i}^{k}(t)e}^{-j\omega t}dt{\int \kappa_{i}^{k}(t)e}^{j\omega t}\right|\\ g_{ij}(\omega )=\sum_{k}\Gamma_{ij}^{k}(\omega )g_{k}{\left(\omega \right)}_{u} \end{array}$$

The convolution kernels mediate the effect of the *k*-th input, at time *t* in the past, on the current response recorded at each channel. In general, they can be regarded as impulse response functions and describe the output at the *i*-th channel, *S*_*i*_(*t*), produced by a spike of the *k*-th exogenous input, *u*_*k*_(*t*). The kernel for each channel obtains analytically from the Jacobian ℑ = ∂*f*/∂*x* describing how the system's hidden neuronal states, *x*(*t*), couple inputs to outputs. For channel *i*, and input *k* the kernel is

(4) $$\begin{array}{c} \kappa_{i}^{k}(\tau )=\frac{\partial s_{i}(t)}{\partial u_{k}\left(t-\tau \right)}\\ =\frac{\partial s_{i}(t)}{\partial g(t)}\frac{\partial g(t)}{\partial x(t)}\frac{\partial x(t)}{\partial x\left(t-\tau \right)}\frac{\partial x\left(t-\tau \right)}{\partial \overset{\cdot }{x}\left(t-\tau \right)}\frac{\partial \overset{\cdot }{x}\left(t-\tau \right)}{\partial u_{k}\left(t-\tau \right)}\\ =L_{i}\frac{\partial g}{\partial x}\exp\left(I\tau \right)I^{-1}\frac{\partial f}{\partial u_{k}} \end{array}$$

This means the kernels are analytic functions of $\overset{\cdot }{x}(t)=f\left(x,u\right)$ and *s*(*t*) = *Lg*(*x*); the network's equations of motion and output function respectively. The use of the chain rule follows from the fact that the only way past inputs can affect current channel outputs is through the hidden states. It is these states that confer memory on the system. In Appendix A, we present an alternative derivation of the cross-spectral density using the Laplace transform of the dynamics in state-space form. This gives a more compact, if less intuitive, series of expressions that are equivalent to the kernel expansion. In this form, the Jacobian is known as the state transition matrix. To accommodate endogenous input delays between different sources and intrinsic transmission delays between different populations within one source, we augment the Jacobian using a Hadamard product; $I\leftarrow {\left(I+\tau I\right)}^{-1}I$, which is based on a Taylor approximation to the effect of delays, τ (see Appendix A.1 of David et al., 2006 for details).

To furnish a likelihood model for observed data-features we include a cross-spectral density *ψ*_*ij*_ induced by channel noise and add a random observation error to the predicted cross-spectral density. Finally, we apply a square root transform to the observed and predicted densities to render the observation error approximately Gaussian. Cross-spectral densities will asymptote to a Wishart distribution at a large sample limit (Brillinger, 1969). However, when averaging each cross or auto-spectral frequency variate across multiple trials, one can appeal to the central limit theorem and assume a near normal distribution. In cases where multiple realisations are limited (see Empirical Demonstration below) the square-root transform renders a Gaussian assumption more valid (see Kiebel et al., 2005 for a comprehensive treatment). The advantage of being able to assume Gaussian errors is that we can invert the model using established variational techniques under something called the Laplace assumption (Friston et al., 2007); this means the current DCM is inverted using exactly the same scheme as all the other DCMs of neurophysiological data we have described.

(5) $$\begin{array}{c} \sqrt{g_{ij}{\left(\omega \right)}_{c}}=\sqrt{g_{ij}(\omega )+\psi {\left(\omega \right)}_{ij}}+ɛ(\omega )\\ \psi {\left(\omega \right)}_{ij}=\{\begin{array}{cc} \psi_{c}+\psi_{s} & i=j\\ \psi_{c} & i\neq j \end{array}\\ \psi_{c}=\alpha_{c}+\beta_{c}/\omega \\ \psi_{s}=\alpha_{s}+\beta_{s}/\omega \end{array}$$

The spectral densities, *ψ*_*c*_ and *ψ*_*s*_ model the contributions of common noise sources (e.g., a common reference channel) and channel-specific noise respectively. As with the neuronal innovations we parameterise these spectral densities as an unknown mixture of white and pink components. The observation error *ɛ* ~ *N*(0, Σ (*λ*)) has a covariance function, Σ(*λ*) = exp(*λ*)*V*(*ω*), where *λ* are unknown hyperparameters and *V*(*ω*) encodes correlations over frequencies^1^.

Eqs. (1) to (5) specify the predicted cross-spectral density between any two channels given the parameters of the observation model {*α*, *β*, *λ*, *θ*} and the neuronal state equations, {*κ*, *H*, *γ*, *A*, *C*}. This means that the cross-spectral density is an analytic function of the parameters *ϑ* = {*α*, *β*, *κ*,*H*, *γ*, *A*, *C*, *λ*,*θ*} and specifies the likelihood *p*(*g*_*c*_ |*ϑ*) of observing any given pattern of cross-spectral densities at any frequency. When this likelihood function is supplemented with a prior density on the parameters, *p*(*ϑ*) (see Moran et al., 2007 and Table 1), we have a full probabilistic generative model for cross-spectral density features *p*(*g*_*c*_,*ϑ*) = *p*(*g*_*c*_ |*ϑ*) *p*(*ϑ*) that is specified in terms of biophysical parameters. Next, we look at how to extract the data features this model predicts.

### Evaluating the cross-spectral density

The assumptions above establish a generative model for cross-spectral features of observed data under linearity and local stationarity assumptions. To invert or fit this model we need to perform an initial feature selection on the raw LFP or M/EEG data. In this section, we describe this procedure, using a vector auto-regression (VAR) model of the multi-channel data and comment briefly on its advantages over alternative schemes. We use a *p*-order VAR-model of the channel data *y*, to estimate the underlying auto-regression coefficients *A*(*p*) ∈ ℜ^*c*^^xc^ (where *c* is the number of channels^2^).

(6) $$y_{n}=A^{\left(1\right)}y_{n-1}+A^{\left(2\right)}y_{n-2}\ldots +A^{\left(p\right)}y_{n-p}+e$$

Here the channel data at the *n*-th time point,*y*_*n*_, represents a signal vector over channels. The autoregressive coefficients *A*^(*k*)^ are estimated using both auto-and cross-time-series components. These, along with an estimated channel noise covariance, *E*_*ij*_ provide a direct estimate of the cross-spectral density, *g*_*ij*_(*ω*)_*c*_ = *f*(A(*p*)), using the following transform:

(7) $$\begin{array}{l} H_{ij}(\omega )=\frac{1}{A_{ij}^{\left(1\right)}e^{iw}+A_{ij}^{\left(2\right)}e^{i2w}+\ldots \ldots +A_{ij}^{\left(p\right)}e^{ipw}}\\ g_{ij}{\left(\omega \right)}_{c}=H{\left(\omega \right)}_{ij}E_{ij}H{\left(\omega \right)}_{ij}^{*} \end{array}$$

The estimation of the auto-regression coefficients, *A*^(*k*)^ ∈ *A*(*p*) uses the spectral toolbox in SPM (http://www.fil.ion.ucl.ac.uk) that allows for Bayesian point estimators of *A*(*p*), under various priors on the coefficients. Details concerning the Bayesian estimation of the VAR-coefficients can be found in Roberts and Penny (2002). Briefly, this entails a variational approach that estimates the posterior densities of the coefficients. This posterior density is approximated in terms of its conditional mean and covariance; *p*(*A*|*y*, *p*) = *N*(*μ*_A_,Σ_A_). These moments are optimised through hyperparameters *v*_*E*_ and *v*_*A*_ (with Gamma hyperpriors; Γ(10^3^, 10^− 3^)) encoding the precision of the innovations *e* and the prior precision, respectively^3^:

(8) $$\begin{array}{l} \mu_{A}=\sum_{A}v_{E}{\tilde{y}}^{T}y\\ \sum_{A}={\left(v_{E}{\tilde{y}}^{T}\tilde{y}+v_{A}I\right)}^{-1} \end{array}$$

Equation 7 uses the posterior mean of the coefficients to provide the cross-spectral density features.

The advantage of our parametric approach is its structural equivalence to the generative model itself. We use uninformative priors but place formal constraints on the estimation of cross-spectral density through the order *p* of the VAR-model. This has important regularising properties when estimating the spectral features. Alternatively, non-parametric methods could be used to quantify the cross-spectral density; e.g., a fast Fourier transform (FFT). However, in the case of *a priori* information regarding model order, several advantages exist for parametric approaches over the conventional FFT. One inherent problem of the FFT is its limited ability to distinguish between signal components at neighbouring frequencies. This resolution in Hertz is roughly reciprocal to the time interval in seconds, over which data are sampled. This is particularly problematic for short time segments where low delta (2–4 Hz) or theta (4–8 Hz) activity may be of interest. Secondly, when long data sequences are evaluated, averaging methods using a windowed FFT must trade-off spectral leakage and masking from side-lobes with broadening in the main lobe, which further decreases resolution. These limitations can be overcome using an AR model since frequencies can be estimated at any frequency point up to the Nyquist rate, and do not require windowing to obtain average steady-state estimates (Kay and Marple, 1981). The principle concern in using these AR methods is frequency splitting (the appearance of a spurious spectral peak), that ensues with overestimation of the model order (Spyers-Ashby et al., 1998). However, we can avoid this problem by exploiting our neural mass model: principled constraints on the order are furnished by the DCM above and follow from the fact that the order of the underlying VAR process is prescribed by the number of hidden neuronal states in the DCM. Heuristically, if one considers a single source, the evolution of its hidden states can be expressed as a *p*-variate VAR(1) process

(9) x(t+τ)=exp(Iτ)x(t)+η(t)

where *η*(*t*) corresponds to exogenous input convolved with the system's kernel. Alternatively, we can represent this process with a univariate AR(*p*) process on a single state. Because there is a bijective mapping between source activity and measurement space, the multivariate data can be represented as a VAR(*p*) process. We provide a formal argument in Appendix B for interested readers.

**Fig. 2:**
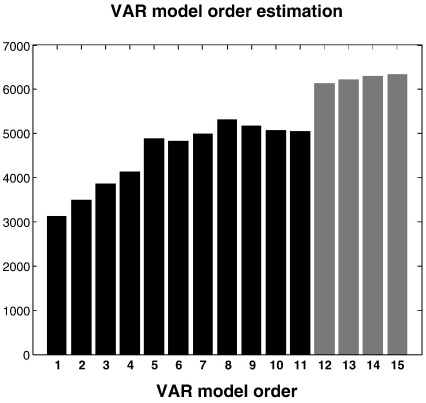
The log-evidence for different order VAR models. The variational Bayes approach described in the text provides the log model evidence for different VAR model orders. This analysis illustrates a large increase in model evidence up to order twelve (black) and small increases thereafter (grey). This increase in evidence occurs at an order that is equal to the number of poles of the DCMs transfer function (see Appendix B).

The number of hidden states per source is twelve (see Fig. 1) and this places an upper bound on the order of the VAR model^4^. The relationship between the VAR model order and the number of hidden states can be illustrated in terms of the log-evidence ln *p*(*y*|*p*) for VAR models with different orders: we convolved a mixture of pink and white noise innovations with the DCM's first-order kernel (using the prior expectations) and used these synthetic data to invert a series of VAR models of increasing order. Fig. 2 shows the ensuing model evidence jumps to a high value when the order reaches twelve, with smaller increases thereafter.

### Model inversion and inference

Model inversion means estimating the conditional density of the unknown model parameters *p*(*ϑ*|*g*_*c*_,*m*) given the VAR-based cross-spectral density features *g*_*c*_ for any model *m* defined by the network architecture and priors on the parameters, *p*(*ϑ*|*m*). These unknown parameters include (i) the biophysical parameters of the neural-mass model, (ii) parameters controlling the spectral density of the neuronal innovations and channel noise, (iii) gain parameters and (iv) hyperparameters controlling the amplitude of the observation error in Eq. (5). The model is inverted using standard variational approaches described in previous publications and summarised in Friston et al., (2007). These procedures use a variational scheme in which the conditional density is optimized under a fixed-form (Laplace) assumption. This optimisation entails maximising a free-energy bound on the log-evidence, 1n *p*(*g*_*c*_ |*m*). Once optimised, this bound can be used as an approximate log-evidence for model comparison in the usual way. Comparing DCMs in a way that is independent of their parameters is useful when trying to identify the most plausible architectures subtending observed responses (Penny et al., 2004; Stephan et al., 2007) and is used extensively in subsequent sections. The focus of this paper is on the approximate log-evidence 1n *p*(*g*_*c*_ |*m*) and conditional densities *p*(*ϑ*|*g*_*c*_,*m*) and, in particular, whether they can support robust inferences on neural-mass models and their parameters.

## Identifiability and face validity

In this section, we try to establish the face validity of the DCM and inversion scheme described in the previous section. Here, we use synthetic datasets generated by models with known parameters. We then try to recover the best model and its parameters, after adding noise to the data. We will address both inference on models and their parameters. This involves searching over a space or set of models to find the model with the greatest evidence. One then usually proceeds by characterising the parameters of the best model in terms of their conditional density. In both inference on models and parameters, we used the same model employed to analyse the empirical data of the next section. This enabled us to relate the empirical results to the simulations presented below.

### Inference on model-space

For inference on models, we generated data from three two-source networks using extrinsic connections from the first to the second source, from the second to the first and reciprocal connections. To assess inference on model-space, we first performed a model comparison using a small set of two source networks, delimited by their forward connections only. Specifically, each of the three models that were used to generate the model-specific data sets, were compared across each set of data. We hope to show that the inversion scheme identified the correct model in all three cases. In all three models exogenous neuronal inputs entered both sources and the connections were all of the forward type. These three models are also evaluated in the empirical analysis. The parameter values for all three models were set to their prior expectations^5^, with the exception of the extrinsic connections, for which we used the conditional estimates of the empirical analysis. Data were generated over frequencies from 4 to 48 Hz and observation noise was added (after the square root transform). The variance of this noise corresponded to the conditional estimate of the error variance from the empirical analysis.

The resulting three data sets were then inverted using each of the three models. For each data set, this provided three log-evidences (one for each model used to fit the spectral data). We normalised these to the log-evidence of the weakest model to produce log-likelihood ratios or log-Bayes factors. The results for the three models are shown in Table 2a. These indicate that, under this level of noise, DCM was able to identify the model that actually generated the data. In terms of inference on model-space, we computed the posterior probability of each model by assuming flat or uniform priors on models; under this assumption *p*(*y*|*m*_*i*_) ∝ *p*(*m*_*i*_|*y*), which means we can normalise the evidence for each model, given one data set and interpret the result as the conditional probability on models. These are expressed as percentages in Table 3b and show that we can be almost certain that the correct model will be selected from the three-model set, with conditional probabilities close to one for correct models and close to zero for incorrect models Following the suggestions of our reviewers, we performed a second analysis where we compared all possible two-source DCM networks. This model space, which comprised 256 models in total, was derived by considering all possible permutations of inputs and connections. We would like to emphasize that this brute force method of testing all possible models (which can be very expensive in terms of computation time) is appropriate only when using small networks with a limited number of free variables. In the applied case of analysing empirical data, DCM is used to test a limited number of hypotheses regarding the type of neuronal architecture that subtends observed experimental responses (e.g. Grol et al., 2007; Stephan et al., 2006a, 2007). This is because (i) the precision of inference with DCM generally favours a strongly hypothesis-driven approach and (ii) the combinatorics of possible DCMs quickly explodes with the number of sources and connections.

**Fig. 3:**
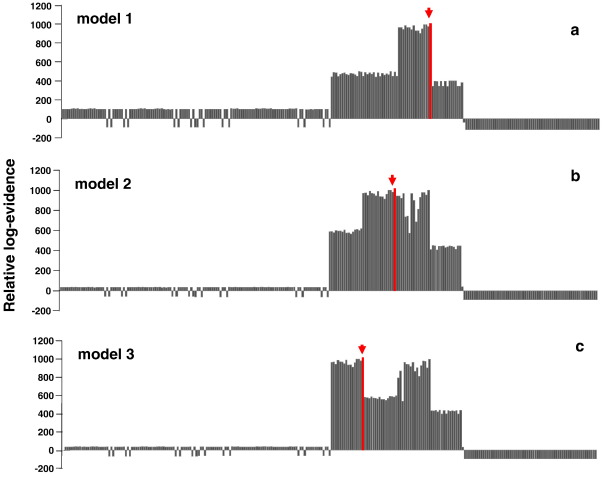
The log-evidence for models tested from three different generative architectures. These are the results of a full test over all possible two-source DCM models, comprising 256 in total. Red bars and arrow indicate the model with the greatest log evidence. In all three cases this corresponds to the correct generative model (a) Generative model 1 comprising forward connections from the first to the second source, (b) Generative model 2 comprising forward connections from the second to the first source and (c) Generative model 3 comprising reciprocal forward connections.

The results of this second analysis show that DCM can correctly identify the generative model, even when all 256 possible models are considered. For each of the three data sets that were inverted, the log-evidence was greatest for the correct generative model (Fig. 3). The relative log-evidence or log Bayes-factors for the best compared to the second best model offered strong support for the correct model, in all three cases (ln *BF*^*1*^ = 14.6 ; ln *BF*^*2*^ = 16.2 ; ln *BF*^*3*^ = 16.4). Note that when we talk of the ‘best' model, we mean a model for which there is strong evidence relative to any competing model. In other words, we can be 95% confident that the evidence for the best model is greater than any other (this corresponds to a relative log-evidence of about three). In summary, Bayesian model comparison with DCM seems to be able to identify these sorts of models with a high degree of confidence.

### Inference on parameter-space

For inference on parameters, we looked at the effects of changing the maximum amplitudes of excitatory postsynaptic potentials (EPSP), which control the efficacy of intrinsic and extrinsic connections and the effects of changing the extrinsic connections themselves. These effects are encoded in the parameters *H*_*e*_ ∈ *ϑ* and *A*^*F*^ ∈ *ϑ*, respectively. We addressed identifiability by inverting a single model using synthetic data with different levels of noise. By comparing the true parameter values to the conditional confidence intervals, under different levels of noise, we tried to establish the accuracy of model inversion and how this depends upon the quality of the data. As above, we chose different levels of noise based upon the error variance estimated using real data. Specifically, we varied the noise levels from 0.001 to 2 times the empirical noise variance, allowing a broad exploration of relative signal-to-noise ratios (SNR) .

**Fig. 4:**
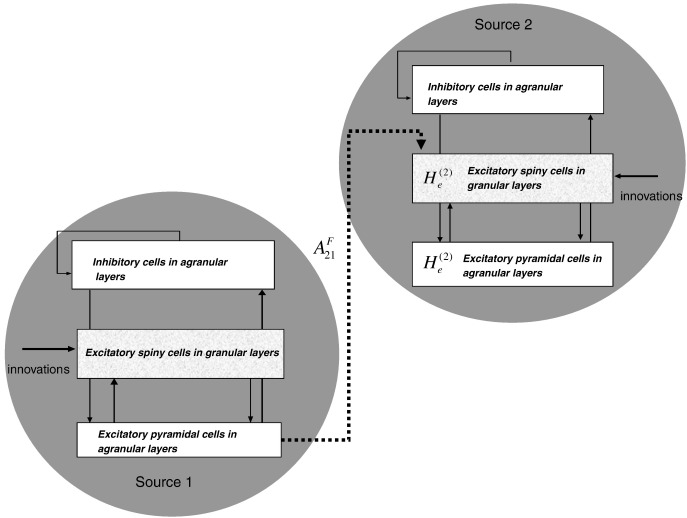
Simulated two source model where excitatory responses are modulated via a scaling of an intrinsic maximum EPSP parameter in source 2: *H_e_^(2)^* and an extrinsic connection from source 1 to source 2: *A*_21_^*F*^. The inversion scheme was tested by recovering the posterior estimates of these parameters, under different levels of observation noise.

The model we used is the same model identified by the empirical analyses of the next section. This model comprised two sources and two LFP channels with no cross-talk between the channels. The parameter values were based on the estimates from the empirical analysis. Specifically, source 1 sent a strong extrinsic connection to source 2, whose excitatory cells had a relatively low postsynaptic response (Fig. 4). All parameter values were set to their prior expectation, except for the parameters of interest *H*_*e*_^(2)^ and *A*_21_^*F*^.

In our DCM, parameters are optimised by multiplying their prior expectation with an unknown log-scale parameter that is exponentiated to ensure positivity. Hence, a log-scale parameter of zero corresponds to a scale-parameter of one, which renders the parameter value equal to its prior expectation. By imposing Gaussian priors on the log-scale parameters we place log-normal priors on the parameters *per se*. To model reduced postsynaptic amplitudes in source 2, *H*_*e*_^2^ had a log-scale parameter of − 0.4 representing a exp(− 0.4) = 67% decrease from its prior expectation. The log-scale parameter encoding the forward connection from source 1 to source 2, namely *A*_2,1_^*F*^, was set to 1.5, representing a exp(1.5) = 448% increase from its prior expectation. Both sources received identical neuronal innovations, comprising white and pink spectral components (as specified in Equation 2 above). Data were generated over frequencies from 4 to 48 Hz.

**Fig. 5:**
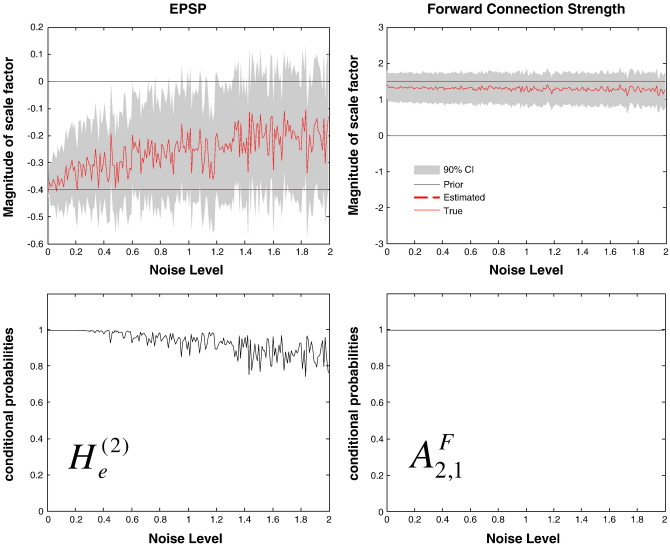
Conditional densities of parameter estimates using the two-source simulations. The data were generated under known parameter values (red line) and mixed with noise (one thousandth to twice the empirical noise estimate). The EPSP parameter (Top left) was exp (− 0.4) = 67% of its prior expectation. The MAP estimates for this log-scale parameter (plotted in hashed red) display a characteristic shrinkage toward the prior of zero at high levels of noise (90% confidence intervals are plotted in grey). The extrinsic connection parameter (Top right) *A*_21_^*F*^ displays a similar behaviour, when simulated at exp(1.5) = 448% of its prior expectation. The grey lines show the prior value (of zero) used for the simulations. The bottom graphs show the conditional probabilities that the MAP estimates of the log-scale parameters differ from their prior expectation.

Posterior density estimates for all parameters, *p*(*ϑ* | *g*_*c*_,*m*) were obtained for 128 intermediate noise levels between one thousandth and twice the empirical noise variance. The conditional expectation or MAP (maximum *a posteriori*) estimates of *H*_*e*_^(2)^ and *A*_2,1_^*F*^ are shown in Fig. 5 (hashed red line). The (constant) true parameter values are indicated by the solid red line, and the prior value is in grey. The shaded areas correspond to the 90% confidence intervals based on the conditional or posterior density. The lower panels show the conditional probabilities *p*(*H*_*e*_^(2)^ < 8) and *p*(*A*_2,1_^*F*^ > 32) that the parameters differed from their prior expectations.

It can be seen that the conditional expectation remained close to the true values for both parameters, despite differences in their conditional precision, which decreased with increasing levels of observation noise. This can be seen in the shrinking Bayesian confidence intervals (grey area) and in the small increase in conditional probabilities with less noise. This effect is more marked for the estimates of *H*_*e*_^(2)^; where the confidence intervals splay at higher noise levels. This jagged variance in the confidence interval itself reflects the simulation protocol, in which each data set comprised a different noise realisation. In addition, the lowest conditional probability (that the parameter posterior estimate differed from the prior) for all simulations, occurred for this EPSP parameter where *p*(*H*_*e*_^(2)^ < 8) = .74 at a high noise level of 1.83. In contrast, the connection strength parameter remained within tight confidence bounds for all noise levels and produced a minimum conditional probability, *p*(*A*_2,1_^*F*^ < 32) = .99. This minimum occurred again as expected, at a high noise levels of 1.72 times the empirical noise level. One can also see, for both parameters a trend for conditional estimates to shrink towards the prior values at higher noise levels; this shrinkage is typical of Bayesian estimators; i.e. when data become noisy, the estimation relies more heavily upon priors and the prior expectation is given more weight (Friston et al., 2003). Importantly, while the 90% confidence bounds generally encompass the true values, the prior values remain outside. In summary, under the realistic levels of noise considered, it appears possible to recover veridical parameter estimates and be fairly confident that these estimates differ from their prior expectation.

## Empirical demonstration

In this section, we present a similar analysis to that of the previous section but using real data. Furthermore, to pursue construct-validity, we invert the model using data acquired under different experimental conditions to see if the conditional estimates of various synaptic parameters change in a way that is consistent with previous analyses of functional connectivity using cross-correlograms. These analyses suggest an increase in coupling between the amygdala and hippocampus that is expressed predominantly in the theta range. This section considers the empirical data set-up, experimental design and inference on models and parameters. We interpret the conditional estimates of the parameters, in relation to the underlying physiology, in the Discussion.

### Empirical LFP data

Local field potential data were acquired from mice (adult male C57B/6J mice, 10 to 12 weeks old) during retrieval of a fear-memory, learned in a Pavlovian conditioning paradigm using acoustic tones (CS+ and CS-) and foot-shock (US). A previous analysis of these data (Seidenbecher et al., 2003) points to the importance of theta rhythms (∼ 5 Hz) during fear-memory retrieval (Pape and Stork, 2003; Buzsaki, 2002). Specifically, Seidenbecher et al., (2003) demonstrated an increase in theta-band coupling between area CA1 of the hippocampus and the lateral nucleus of the amygdala (LA) during presentation of the CS+. Moreover, theta synchrony onset was correlated with freezing, a behavioural index of fear-memory (Maren et al., 1997). For the purposes of demonstrating our DCM, we here revisit the data of a single animal and show that this ‘on/off’ theta synchrony can be explained with plausible neurobiological mechanisms at the synaptic level, using the methodology described in the previous sections. These data represent quasi-stationary signals as evidenced by small time variations in signal strength (Figs. 5a and b). The term “steady-state” refers to the frequency estimates that represent only the constant spectral amplitude and are the complete data feature captured by this DCM. Below, we examine induced steady-state responses, where spectral estimates are averaged over independent trials. However, there is no principled reason why the current model may not be inverted using spectra from a time-frequency analysis of evoked responses or event related responses, under the assumption of local stationarity over a few hundred milliseconds (e.g. Robinson et al., 2008; Kerr et al., 2008).

**Fig. 6:**
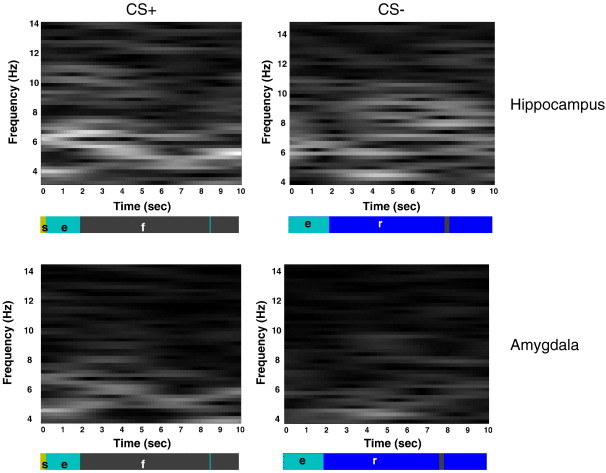
CS+ (Left) and CS- (Right) spectrograms. Time-frequency data demonstrating theta activity at hippocampal (Top) and amygdala (Bottom) electrodes during the CS+ and CS-. These plots are scaled relative to the maximum theta peak in the CS+ hippocampal image. They are displayed with corresponding behavioural modes represented as colour-bars; where ‘f’ demarks freezing periods (the behavioural correlate of fear recall), ‘e’ exploration, ‘r’ risk assessment and ‘s’ stereotypical behaviour. During the CS+ condition theta activity can be observed in both electrodes, in contrast, during the CS- condition, theta activity is evident in hippocampal data but much less in the amygdala.

**Fig. 7:**
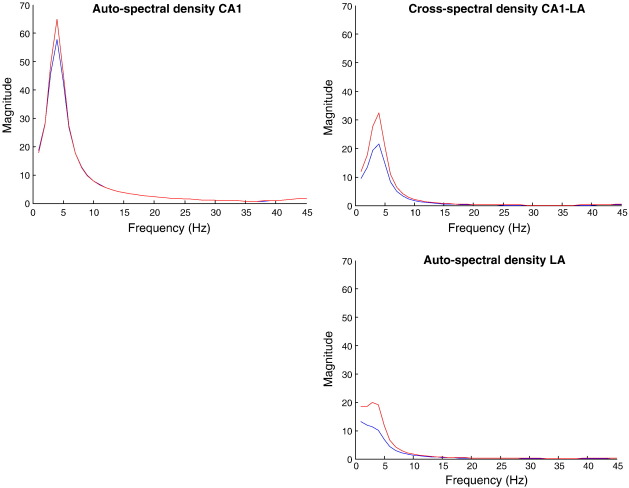
Average cross-spectral densities across all CS+ (red) and CS- (blue) trials. Top left: hippocampal autospectrum, Top right: hippocampal-amygdala cross spectrum, Bottom right: amygdala autospectrum. These spectral data features were evaluated from three second epochs after the first freezing behaviour during CS+ and the time/order matched CS- trials. Peaks at theta frequency are evident in both CS+ and CS- conditions with reduced theta activity in the amygdala during CS-.

LFP data were recorded from two electrodes in the LA and the CA1 of the dorsal hippocampus. The data comprised 6 min of recording, during which four consecutive CS- tones and four consecutive CS+ tones were presented, each lasting 10 s. Freezing behaviour was seen prominently during the CS+. Preliminary analysis, using time-frequency spectrograms, revealed that the hippocampal region exhibited strong background theta rhythms, during CS+ and CS- epochs (Figs. 5a and b); whereas theta activity in lateral amygdala was prominent only during the CS+ stimulus. Fig. 6 displays the first CS+ and CS- epochs of fear recall. Cross-spectra were computed for three-second epochs that followed the onset of freezing behaviour in the four CS+ epochs and order-time matched CS- epochs. Cross-spectral densities were computed from 4 to 48 Hz, using an eighth-order VAR model, for each epoch and averaged across conditions (Fig. 7). This revealed spectral features that corroborated the analysis of Seidenbecher et al., (2003); with pronounced fast theta activity in the hippocampus and a marked theta peak in the cross-spectral density. The amygdala showed a broader spectrum, with a preponderance of lower theta activity and a theta peak in, and only in, the CS+ trial.

### Dynamic causal modelling

These cross-spectral densities were then inverted using a series of generative models. These models were used to test the direction of information flow during heightened theta synchrony following CS+. Given key experimental differences between CS- and CS+ trials, we introduced log-scale parameters *β*_*ki*_ to model trial-specific variations in specified parameters:

(10) $$\begin{array}{l} \vartheta_{i}^{j}=\vartheta_{i}\exp\left(\sum_{k}X_{jk}\beta_{ki}\right)\\ X=\left[\begin{array}{c} 0\\ 1 \end{array}\right] \end{array}$$

*β*_*ki*_ is the *k*-th experimental effect on the *i*-th parameter and *ϑ_i_^j^* is the value of the *i*-th parameter *ϑ*_*i*_ in the *j*-th trial or condition. These effects are meditated by an experimental design matrix *X*, which encodes how experimental effects are expressed in each trial.

Eq. (10) is a generic device that we use to specify fully parameterised experimental effects on specific parameters in multi-trial designs. In this example, *β*_1*i*_ is simply a log-scale parameter (Table 1) specifying the increase (or decrease) in CS+ relative to CS- trials. The parameters showing trial-specific effects were the extrinsic connections and excitatory post synaptic amplitudes; all other parameters we fixed over trials.

#### Inference on models

**Fig. 8:**
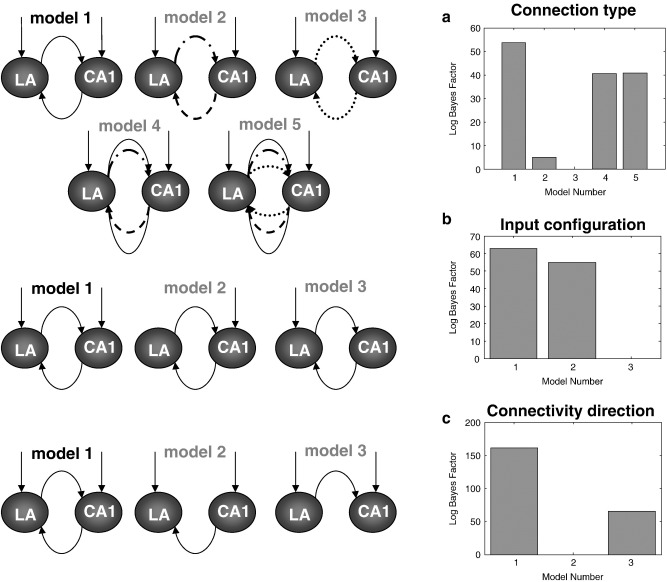
Results of the Bayesian model comparison. Log Bayes factors are plotted relative to the worst model in each comparison. (a) Optimal connection type is found in Model 1, where the connections are of the ‘forward’ type. (b) Model evidence supports Model 1, where exogenous inputs enter both the hippocampus and amygdala. (c) Model evidences suggest reciprocal connections between the hippocampus and amygdala.

The extrinsic connection types in our DCM are based on connections between isocortical areas (Felleman and Van Essen 1991); however, in this analysis we are dealing with allocortical (CA1) and subcortical (LA) brain regions that have no clearly defined hierarchical relationship. Therefore, our first step was to establish which connection type best explained the measured LFP data. We approached this using model comparison using DCMs with reciprocal connections between CA1 and LA. The connections in these models were (model 1) forward; (model 2) backward; (model 3) lateral; (model 4) a combination of forward and backward and (model 5) a combination of all three. Bayesian model comparison based on the log-evidence indicated that the most likely type of inter-regional connections was of the ‘forward’ type (model 1); where connections originate from pyramidal cells and target excitatory interneurons. Fig. 8a shows the relative model evidences for the five models (i.e., the log-Bayes factor with respect to the worst model).

Next, employing the optimal connection type, three different input schemes were tested to find where driving inputs, i.e. from cortical regions, enter during CS+ and CS- epochs. These DCM's included; (model 1) comprising exogenous inputs to both CA1 and LA; (model 2) exogenous input to hippocampal region CA1 only and (model 3) the lateral amygdala only. Fig. 8b shows that the best model is model 1; where inputs enter both the lateral amygdala and hippocampal CA1.

Having established a causal architecture for the inputs, three further models were tested to examine whether connections were bidirectional or unidirectional. These results are displayed in Fig. 8c, where model 1 had bidirectional connections, model 2 had unidirectional hippocampal to amygdala connections and model 3 had connections from amygdala to hippocampus. We see that the most plausible model contains bidirectional connections between hippocampus and amygdala.

**Fig. 9:**
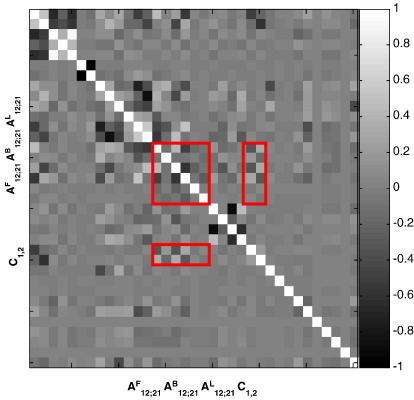
Posterior Correlation matrix of the DCM for the empirical data set. Data from a DCM comprising all forward, backward and lateral connections as well as inputs to both sources was used to demonstrate minimal posterior correlations in the set of parameters comprising the hierarchical search. Red boxes highlight the correlations among these parameters. The mean of the absolute value of correlations within this set was − 0.24.

**Fig. 10:**
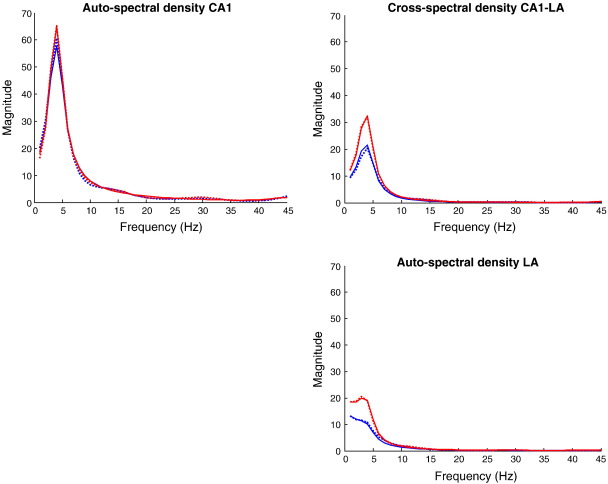
Model fits for all empirical data (CS+ : red, CS-: blue). Top left: hippocampal autospectrum, Top right: hippocampal-amygdala cross spectrum, Bottom right: amygdala autospectrum. The measured spectra are shown with a dashed line and the conditional model predictions with a full line.

In principle, as in the analysis of synthetic data above, there are 256 possible DCMs that could explain the empirical data. However, to provide an exemplar strategy for when where exhaustive model searches are not possible, we finessed the search of model space by optimising various model attributes sequentially. This series of line searches can be regarded as a heuristic search over model space to identify the most likely model. One concern in using this sort of heuristic search is that conditional dependencies among the free-parameters do not guarantee the global maximum is found. To address this, we performed a further analysis of the ‘complete’ model, which comprised reciprocal connections of all types (forward and backward and lateral), and inputs to both regions. The resulting conditional covariance matrix was examined in order to investigate potential co-dependencies between the parameters. The posterior correlation matrix is shown in Fig. 9 and shows only relatively small interdependencies between the search parameters. Overall, the accuracy of the best performing model was impressive; the fits to the cross-spectral data or shown in Fig. 10 and are almost indistinguishable from the observed spectra. Having identified this model we now turn to inference on its parameters.

#### Inference on parameters

**Fig. 11:**
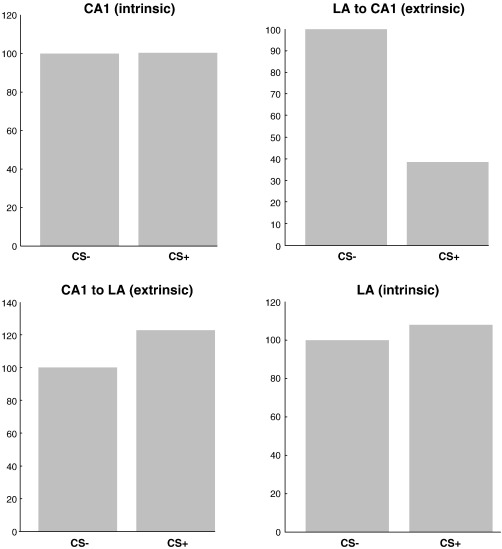
Trial-specific effects encoding differences between the CS+, relative to CS- trials. Top left: Hippocampal EPSP displays < 1% change on CS+ trials. Top right: amygdala to hippocampus forward connection strength decreases by 72% on CS+ trials. Bottom left: Hippocampus to amygdala forward connection strength increases by 26% on CS+ trials. Bottom right: amygdala EPSP increases by 8% in CS+ relative to CS- trials.

We now look at the conditional probabilities of key parameters showing trial-specific or conditioning effects, under the most plausible model. These parameters were the extrinsic connection strengths and intrinsic postsynaptic efficacies. When comparing the CS- and CS+ trials, we observe decreased amygdala-hippocampal connectivity and increased hippocampal-amygdala connectivity. Fig. 11 shows the MAP estimates of ln *β*_1*i*_, which scale the extrinsic connections relative to 100% connectivity in CS-. In addition, there were small increases in postsynaptic efficacy in the amygdala for the CS+ relative to CS- Quantitatively, hippocampus-amygdala connectivity increased by 26%, with a conditional probability of 99.97% that this effect was greater than zero. In contrast, amygdala-hippocampus forward connections decreased by 72%, with a conditional probability of almost one. The relative change of intrinsic amygdala excitatory postsynaptic amplitude was 8% with a high conditional probability 99.85% that the increase was greater than zero. In contrast, changes in hippocampal excitatory postsynaptic amplitude were unremarkable, (0.002%) and with a conditional probability that was close to chance (69.70%).

In summary, these results suggest that the hippocampus and amygdala influence each other through bidirectional connections. Steady states responses induced by CS+, relative to CS- stimuli appear to increase the intrinsic sensitivity of postsynaptic responses in the amygdala and with an additional sensitization to extrinsic afferents from the hippocampus. At the same time the reciprocal influence of the amygdala on the hippocampus is suppressed. These conclusions are exactly consistent with early hypotheses based on correlations (see below).

## Discussion

We have described a dynamic causal model (DCM) of steady-state responses that are summarised in terms of cross-spectral densities. These spectral data-features are generated by a biologically plausible, neural-mass model of coupled electromagnetic sources. Under linearity and stationarity assumptions, inversion of the DCM provides conditional probabilities on both the models and the synaptic parameters of any particular model. The model employed here has previously been shown to produce oscillatory activity at all standard EEG frequency bands, in its linear approximation (Moran et al., 2007). A nonlinear model analysis could uncover interesting dynamics in some of these bands and will be the subject of further research. This would call for a relaxation of the linearization assumption and present an interesting challenge for model inversion (*c.f*., Valdes et al., 1999).

Recently, a number of studies have established the utility neural mass models for interrogating EEG data. The motivations behind this approach are varied. In Riera et al., (2006) neural masses are used to investigate local electrovascular coupling and their multi-modal time domain expression in EEG and fMRI data; while Valdes et al. (1999) employ neural masses to examine the emergent dynamic properties of alpha-band activity. Closer to the work presented here, Robinson et al., (2004) have developed a frequency domain description of EEG activity that highlights the importance of corticothalamic interactions, using neural field models. As in Robinson et al., (2004), the goal of DCM for steady-state responses is to make inferences about, regionally-specific neurotransmitter and neuromodulatory action that unfolds in a connected but distributed network. The DCM presented in this paper assumes a network of point sources (c.f., equivalent current dipoles) that may be usefully extended to cover neural field models of the sort considered by Robinson et al., (2004). DCM enables inference about synaptic physiology and changes induced by pharmacological or behavioural manipulations both within and between neural ensembles; furthermore, the methodology can be applied to the cross-spectral density of invasive or non-invasive electrophysiological recordings.

Usually, in Dynamic Causal Modelling, data prediction involves the integration of a dynamical system to produce a time-series. In the current application, the prediction is over frequencies; however, the form of the inversion remains exactly the same. This is because in DCM for deterministic systems (i.e., models with no system or state noise) the time-series prediction is treated as a finite-length static observation, which is replaced here with a prediction over frequencies. The only difference between DCM for time-series and DCM for cross-spectral density is that the data-features are represented by a three dimensional array, covering *c* × *c* channels and *b* frequency-bins. In conventional time-series analysis the data-features correspond to a two-dimensional array covering *c* channels and *b* time-bins. The spectral summary used for data inversion comprises the magnitude of cross-spectra, which is a sufficient data-feature, under quasi-stationarity assumptions. Information regarding instantaneous phase or phase-coupling among sources are not considered in this treatment. In some settings, phase-coupling has been used in linear and nonlinear settings to model information exchange across discrete brain sources (e.g., Brovelli et al., 2004, Rosenblum et al., 1996). The DCM presented here represents a complement to this approach by offering a biophysically meaningful, mechanistic description of neuronal interactions. An alternative DCM approach for M/EEG analysis has been developed to describe (time-dependent) phenomenological coupling among frequencies at different brain sources that occur through both linear and nonlinear mechanisms (Chen et al., 2008). However, neither DCM model the instantaneous phase. Other recent developments in M/EEG data analysis have tackled this issue: Approaches involving ICA (Anemüller et al., 2003) have been used to describe the phases of induced responses on a trial by trial basis, and make use of complex lead-field distributions to retain the imaginary parts of the source signals at the scalp level. However this approach studies independent components of brain activity and as such, is not directly comparable to DCM. DCM for phase responses is an active area of research (Penny et al., 2008) and will receive a full treatment elsewhere.

Our simulation studies provide some face validity for DCM, in terms of internal consistency. DCM was able to identify the correct model and, under one model, parameter values were recovered reliably in settings of high observation noise. Changes in the postsynaptic responsiveness, encoded by the population maximum EPSP, were estimated veridically at levels below prior threshold, with a conditional confidence of more than 74%; even for the highest levels of noise. Similarly, inter-area connection strength estimates were reasonably accurate under high levels of noise. With noisy data, parameter estimates tend to shrink towards their prior expectation, reflecting the adaptive nature of the weights afforded to prior and data information in Bayesian schemes.

We have presented an analysis of empirical LFP data, obtained by invasive recordings in rat CA1 and LA during a fear conditioning paradigm. A previous analysis of these data (Seidenbecher et al., 2003) showed prominent theta band activity in CA1 during both CS+ and CS- conditions, whereas LA expresses significant theta activity during CS+ trials only. Using an analysis of functional connectivity^6^, based on cross-correlograms of LA/CA1 activity in the theta range, Seidenbecher et al., (2003) demonstrated an increase in connectivity between these two brain regions during CS+ trials. This is consistent with a trial-specific enabling or gating of the CA1 → LA connection during retrieval of conditioned fear in the CS+ condition, leading to a transient coupling of LA responses to the condition-independent theta activity in CA1. However, this analysis of functional connectivity was unable to provide direct evidence for directed or causal interactions. This sort of evidence requires a model of effective connectivity like DCM. The DCM analysis in the present study confirmed the hypothesis based on the cross-correlogram results of Seidenbecher et al., (2003). The DCM analysis showed a selective increase in CA1 → LA connectivity during CS+ trials, accompanied by a decrease in LA → CA1 connection strength. An additional finding was the increase in the amplitude of postsynaptic responses in LA during CS+ trials. This result may represent the correlate of long term potentiation of LA neurons following fear conditioning (Rodrigues et al., 2004; LeDoux, 2000). In summary, one could consider these results as a demonstration of construct validity for DCM, in relation to the previous analyses of functional connectivity using cross-correlograms.

The analysis of parameter estimates was performed only after Bayesian model selection. In the search for an optimum model, we asked (i) which connection type was most plausible, (ii) whether neuronal inputs drive CA1, LA or both regions; and (iii) which extrinsic connectivity pattern was most likely to have generated the observed data (directed CA1 → LA or LA → CA1 or reciprocal connections). The results of sequential model comparisons showed that there was a very strong evidence for a model in which (i) extrinsic connections targeted excitatory neurons, (ii) neuronal inputs drove both CA1 and LA and (iii) the two regions were linked by reciprocal connections. While there is, to our knowledge, no decisive empirical data concerning the first two issues, the last conclusion from our model comparisons is supported strongly by neuroanatomic data from tract-tracing studies. These have demonstrated prominent and reciprocal connections between CA1 and LA (see Pitkänen et al., 2000 for a review). This correspondence between neuroanatomic findings and our model structure, which was inferred from the LFP data, provides further construct validity, in relation to neuroanatomy.

In conclusion, this study has introduced a novel variant of DCM that provides mechanistic explanations, at the level of synaptic physiology, for the cross-spectral density of invasive (LFP) or non-invasive (EEG) electrophysiological recordings. We have demonstrated how this approach can be used to investigate hypotheses about directed interactions among brain regions that cannot be addressed by conventional analyses of functional connectivity. A previous (single-source) DCM study (Moran et al., 2008) of invasive LFP recordings in rats demonstrated the consistency of model parameter estimates with concurrent microdialysis measurements. The current study is another step towards establishing the validity of models, which we hope will be useful for deciphering the neurophysiological mechanisms that underlie pharmacological effects and pathophysiological processes (Stephan et al., 2006b).

## Software note

Matlab routines and demonstrations of the inversion described in this paper are available as academic freeware from the SPM website (http://www.fil,ion.ucl.ac.uk/spm) and will be found under the ‘api_erp’, ‘spectral’ and ‘Neural_Models’ toolboxes in SPM8.

## Figures and Tables

**Table 1:** Parameter Priors for model parameters including the observation model, neuronal sources, and experimental effects

Parameter ϑ*_i_ *= π_*i*_exp(Θ_*i*_)	Interpretation	Prior	
		Mean: π_*i*_	Variance: Θ_*i*_ = *N*(0,*σ*_*i*_)
*Observation model*			
*α*_*u*_	Exogenous white input	*π*_*α*___*u*__ = 0	*σ*_*α*___*u*__ = 1/16
*α*_*s*_	Channel specific white noise	*π*_*α*___*s*__ = 0	*σ*_*α*___*s*__ = 1/16
*α*_*c*_	White noise common to all channels	*π*_*α*___*c*__ = 0	*σ*_*α*___*c*__ = 1/16
*β*_*u*_	Exogenous pink input	*π*_*β*___*s*__ = 0	*σ*_*β*___*u*__ = 1/16
*β*_*s*_	Channel specific pink noise	*π*_*β*___*c*__ = 0	*σ*_*β*___*s*__ = 1/16
*β*_*c*_	Pink noise common to all channels	*π*_*θ*___*i*__ = 1	*σ*_*θ*___*i*__ = exp(8)
*θ*_1…*s*_	Lead-field gain	*π*_*λ*_ = 0	*σ*_*λ*_ = 1
*λ*	Noise hyperparameter		

*Neuronal sources*			
*κ*_*e*__/*i*_	Excitatory/inhibitory rate constants	*π*_*κ*___*e*__ = 4 *ms*^− 1^*π*_*κ*___*i*__ = 16 *ms*^− 1^	*σ*_*κ*___*e*__ = 1/8 *σ*_*κ*___*i*__ = 1/8
*H*_*e*__/*i*_	Excitatory/inhibitory maximum post-synaptic potentials	*π*_*H*___*e*__ = 8 *mV π*_*H*___*i*__ = 32 *mV*	*σ*_*H*___*e*__ = 1/16 *σ*_*H*___*i*__ = 1/16
*γ*_1,2,3,4,5_	Intrinsic connections	*π*_*γ*___1__ = 128	*σ*_*γ*___1__ = 0
*π*_*γ*___2__ = 128	*σ*_*γ*___2__ = 0
*π*_*γ*___3__ = 64	*σ*_*γ*___3__ = 0
*π*_*γ*___4__ = 64	*σ*_*γ*___4__ = 0
*π*_*γ*___5__ = 4	*σ*_*γ*___5__ = 0
*A*^*F*^	Forward extrinsic connections	*π*_*A*^*F*^_ = 32	*σ*_*A*^*F*^_ = 1/2
*A*^*B*^	Backward extrinsic connections	*π*_*A*^*B*^_ = 16	*σ*_*A*^*B*^_ = 1/2
*A*^*L*^	Lateral extrinsic connections	*π*_*A*^*L*^_ = 4	*σ*_*A*^*L*^_ = 1/2
*C*	Exogenous input	*π*_*C*_ = 1	*σ*_*c*_ = 1/32
*d*_*i*_	Intrinsic delays	*π*_*d*___*i*__ = 2	*σ*_*d*___*i*__ = 1/16
*d*_*e*_	Extrinsic delays	*π*_*d*___*e*__ = 10	*σ*_*d*___*e*__ = 1/32
Design *β*_*ki*_	Trial specific changes	*π*_*β*___*ki*__ = 1	*σ*_*β*___*ki*__ = 1/2

In practice, the non-negative parameters of this model are given log-normal priors, by assuming a Gaussian density on a scale parameter, Θ_*i*_ = *N* (0,*σ*_*i*_), where *ϑ*_*i*_ = *π*_*i*_exp(Θ_*i*_), and *π*_*i*_ is the prior expectation and *σ*_*i*_^2^ is its log-normal dispersion.

**Table 2a:** Inference on model space: results of the Bayesian inversion on data simulated using three different network architectures (column-wise)

Simulated network connections	*A_2,1_^F^*	*A_1,2_^F^*	*A_2,1_^F^* and *A_1,2_^F^*
Modelled connections			
*A_2,1_^F^*	**416.6**	0	0
*A_1,2_^F^*	0	**399.2000**	0.5000
*A_2,1_^F^* and *A_1,2_^F^*	398.4	381.6000	**561.2000**

Log-Bayes factors are presented relative to the worst model for each network. Best performing models are in bold. For all three simulations, the corresponding model-architecture was found to have the highest Log-Bayes factor.

**Table 2b:** Inference on model space: Posterior probabilities of each model are computed by assuming flat or uniform priors on models; normalising these values gives the conditional probability of the models presented here as percentages

Simulated network connections	*A_2,1_^F^*	*A_1,2_^F^*	*A_2,1_^F^* and *A_1,2_^F^*
Modelled connections	%		
*A_2,1_^F^*	100	0	0
*A_1,2_^F^*	0	100	0
*A_2,1_^F^* and *A_1,2_^F^*	0	0	100

## Appendix A. Laplace description of cross-spectral density

Consider the State Space Model for a particular neuronal source

$$\begin{array}{l} \overset{\cdot }{x}=Ax+Bu\\ y=Cx+Du \end{array}$$

where *A* is the state transition matrix or Jacobian, *x* are the hidden states (cf. Eq. (1)) and *y* is the source output. The Laplace transform gives

(AI.1) $$\begin{array}{c} sX(s)=AX(s)+BU(s)\\ Y(s)=CX(s)+DU(s)\\ \Rightarrow \\ X(s)={\left(sI-A\right)}^{-1}BU(s)\\ \Rightarrow \\ Y(s)=\left(C{\left(sI-A\right)}^{-1}B+D\right)U(s)\\ =H(s)U(s) \end{array}$$

Evaluating at *s* = *jω* gives the frequency output of the system. Given that the cross-spectrum for two signals *i* and *j* is defined as *S*_*ij*_ = *Y_i_Y_j_*^⁎^ and that inputs to the system are seen by both sources, we can write the output cross-spectral density as

(AI.2) $$S_{ij}=H_{i}H_{j}^{*}\left|U\right|$$

where *H*_*i*_ is computed from the transition matrices of each source directly. Furthermore, assuming white noise input we see from

(AI.3) $$\begin{array}{c} y(t)=F^{-1}\left(H\left(j\omega \right)\right)F^{-1}\left(U\left(j\omega \right)\right)\\ F^{-1}\left(U\left(j\omega \right)\right)=\delta (t) \end{array}$$

that *H*_*i*_ are the Fourier Transforms of the impulse responses. In our model, we supplement the input with pink (*1/f*) noise to render the input biologically plausible input. We can now see directly how the cross-spectral density in Eqs. (A1.2) and (3) are equivalent, in terms of system response to the unit impulse.

### Appendix B. VAR model order selection from the number of hidden states

Consider the discrete-time signal described by the difference equation

(AII.1) $$y(t)=-a_{1}y\left(t-1\right)-a_{2}y\left(t-2\right)\ldots -a_{p}y\left(t-p\right)+ɛ$$

The Laplace transform of a sampled signal is known as the Z-transform

(AII.2) $$\begin{array}{c} L\left(y(t)\right)=\sum_{n=0}^{\infty }y\left[n\right]\int_{0^{-}}^{\infty }\delta \left(t-nT\right)e^{-st}\\ Y(z)=\sum_{n=0}^{\infty }y\left[n\right]e^{-st} \end{array}$$

For the AR model of AII.1 we obtain a Z domain representation

(AII.3) $$Y(z)=-a_{1}z^{-1}Y(z)-a_{2}z^{-2}Y(z)\ldots a_{p}z^{-p}Y(z)+ɛ(z)$$

Now consider again the state-space form of each source in Eq. (AI.1). We see that the form of *H*(*s*) is a polynomial quotient, where the denominator is the characteristic polynomial of the Jacobian *A*. This contains powers of *s* up to the number of columns in *A*, indexed by the number of hidden states; i.e. the length of vector *x.* Hence, for *q* roots by partial fraction expansion we obtain

(AII.4) $$H(s)=\frac{A}{s-\lambda_{1}}+\frac{B}{s-\lambda_{2}}+\ldots \frac{K}{s-\lambda_{q}}$$

Using the s–z relation *s* + *β* = 1 – *z*^− 1^*e*^− ^*^βT^*, we obtain the order of the AR model *p*, determined by the number of roots of the Jacobian *q* to give the delay *z*^− *p*^ in Eq. (AII.3).

## Footnotes

1 In our work, we use an AR(1) autoregression model of errors over frequencies, with an AR coefficient of one half and ensure that the error covariance components associated with the cross-spectral density between channels *i* and *j* are the same as the corresponding component for the cross-spectral density between channels *j* and *i*.

2 For computational expediency, if there are more than eight channels, we project the data and predictions onto an eight-dimensional subspace defined by the principal components of the prior covariance matrix in channel space

$$\sum_{i}\frac{\partial L}{\partial \theta_{i}}\sigma_{i}^{2}\frac{\partial L^{T}}{\partial \theta_{i}}$$

where *σ_i_^2^* is the prior variance of the *i*-th spatial or gain parameter.

3 $\tilde{y}$comprise the time lagged data.

4 In practice, we do not use the upper bound but use *p* = 8 for computational expediency; this seems to give robust and smooth spectral features.

5 These expectations are biologically plausible amplitudes and rate constants that have been used in previous instances of the model (Jansen et al., 1993; David et al., 2005) and are summarized in Moran et al., 2007 and Table 1. In this study, prior variances on the intrinsic connectivity parameters were set to zero.

6 Functional connectivity is defined as the statistical dependence between two biophysical time-series, whereas effective connectivity refers to the directed and casual influence one biophysical system exerts over another (Friston et al., 2003)
